# Fibronectin Assembly in the Crypts of Cytokinesis-Blocked Multilobular Cells Promotes Anchorage-Independent Growth

**DOI:** 10.1371/journal.pone.0072933

**Published:** 2013-08-12

**Authors:** Rajesh Kumar Gupta, Staffan Johansson

**Affiliations:** Department of Medical Biochemistry and Microbiology, Uppsala University, Uppsala, Sweden; University of Science and Technology of China, China

## Abstract

Anchorage-independent growth is a characteristic feature of cancer cells. However, it is unclear whether it represents a cause or a consequence of tumorigenesis. For normal cells, integrin-mediated adhesion is required for completion of the G1 and cytokinesis stages of the cell cycle. This study identified a mechanism that can drive anchorage-independent growth if the G1 checkpoint is suppressed. Cells with defective G1 checkpoint progressed through several rounds of the cell cycle in suspension in spite of uncompleted cytokinesis, thereby forming bi- and multilobular cells. Aurora B and CEP55 were localized to midbodies between the lobes, suggesting that the cytokinesis process reached close to abscission. Integrin-mediated re-attachment of such cells induced cytokinesis completion uncoupled from karyokinesis in most cells. However, a portion of the cells instead lost the constriction and became binucleated. Also, long-term suspension culture in soft agar produced colonies where the cytokinesis block was overcome. This process was fibronectin-dependent since fibronectin-deficient cells did not form colonies unless fibronectin was expressed or exogenously added. While fibronectin normally is not deposited on non-adherent single cells, bi/multilobular cells accumulated fibronectin in the intussusceptions. Based on our data we conclude: 1) Suppression of the G1 checkpoint allows multiple rounds of the cell cycle in detached cells and thereby enables matrix formation on their surface. 2) Uncompleted cytokinesis due to cell detachment resumes if integrin interactions are re-formed, allowing colony formation in soft agar 3) Such delayed cell division can generate binucleated cells, a feature known to cause chromosomal instability.

## Introduction

During tumorigenesis cells acquire the ability to survive and proliferate under non-adherent conditions. Anchorage-independent growth (AIG) in soft agar is considered to be the *in vitro* assay, which best correlates with assays for tumor growth *in vivo* [1,2]. However, anchorage-independent cells do not always generate tumors in animal models [3]. The reason for this variability and the mechanisms underlying AIG are still poorly understood.

Anchorage-dependent cells kept in suspension culture have been shown to accumulate either late in the G1 phase or in the cytokinesis phase, and these two anchorage-regulated cell cycle stages were concluded to prevent AIG [4]. Anchorage-independent cells often have intrinsic genetic defects that overcome these two cell cycle blocks, but some cells depend on external factors in the culture medium to grow anchorage-independently [5–7]. Anchorage-dependent passage through the G1 phase involves regulation of the retinoblastoma protein, the CDK inhibitors p21/p27, and cyclin D- and cyclin E-dependent kinases by co-operating signals from growth factor receptors and integrins [8–12]. Less is known regarding the adhesion-dependent mechanisms that regulate the cytokinesis process and their role in AIG. Integrin trafficking [13] and unidentified ECM-integrin signals [4,14] have been shown to be required for cytokinesis, while growth factor signals apparently are dispensable [12]. Also, constitutively active Ras was found to override the cytokinesis arrest in suspension cultures [4]. The cytokinesis block has been suggested to be a protective mechanism against tumorigenesis, e.g. if the G1 checkpoint would be suppressed by somatic mutations or virus infections. Such cells were predicted to accumulate either as multinucleated giant cells or as binucleated cells, depending on whether the cell cycle would continue or not [4,12]; in both cases the cells were assumed to be non-proliferating.

Since there is no strict correlation between AIG and tumorigenic potential we investigated the possibility that there are differences in the regulation of the suspension-induced G1 block and/or the cytokinesis block in different anchorage-independent cells. In this study, we found that cells with a suppressed G1 block actually can overcome the suspension-induced cytokinesis block. This becomes possible as a consequence of two processes: 1) the continued progression of the cell cycle although cytokinesis is uncompleted and 2) the subsequent fibronectin (FN) polymerization. Permissive conditions for FN deposition are provided by the intussusceptions of lobular-shaped cells formed due to the cytokinesis block. Thereby integrin signals are generated which eventually allow abscission and completion of cytokinesis; however, this occurs uncoupled from mitosis. Based on our data we present a model where alternative mechanisms regulate AIG, and where one of them is a previously not recognized potential cause of cancer by linking suppression of G1 checkpoint to chromosomal instability.

## Results

### Cells with defective suspension-induced G1 block form colonies in soft agar in spite of functional suspension-induced cytokinesis block

**Figure 1:**
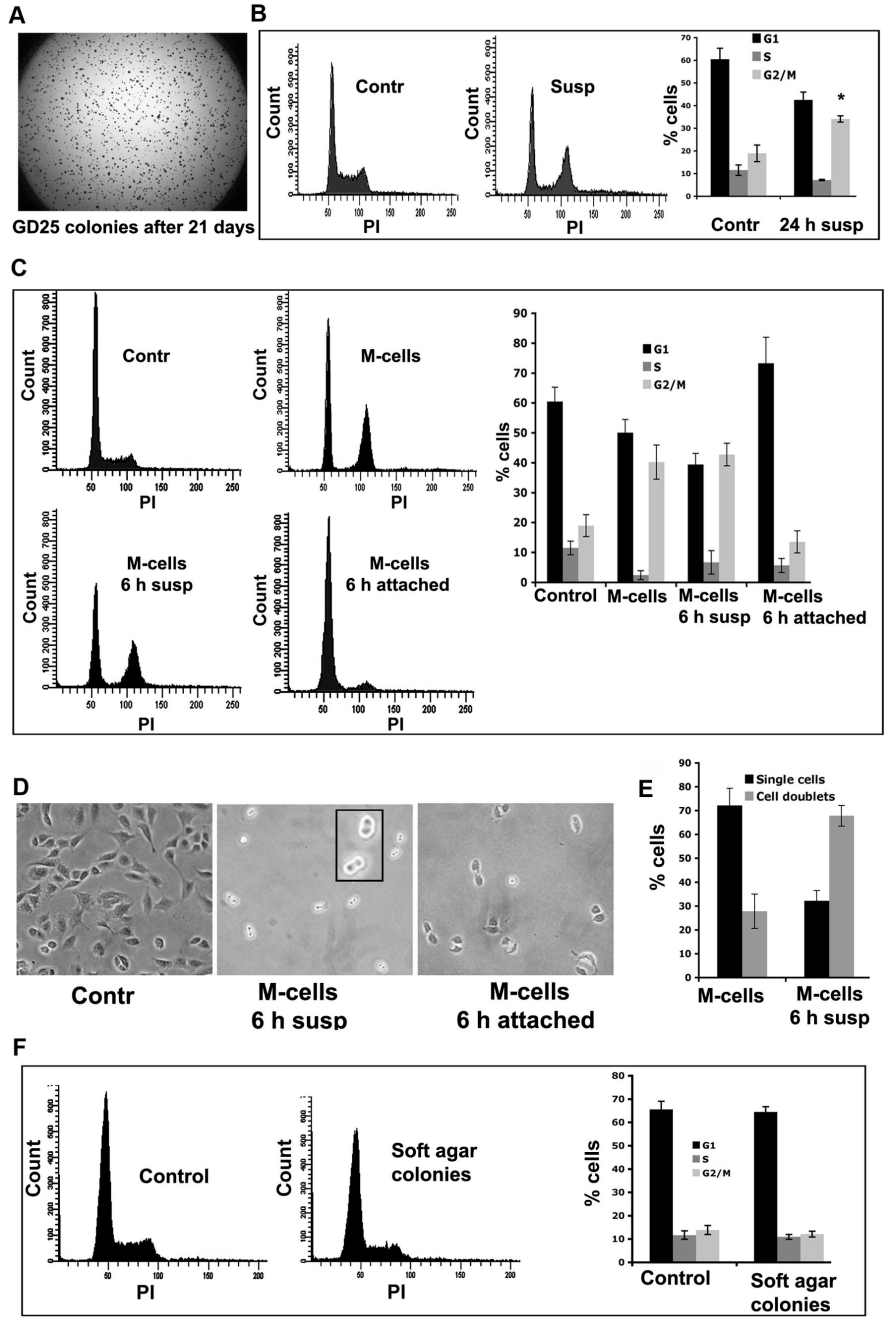
GD25 cells have functional cytokinesis-block and form colonies in soft agar. (**A**) GD25 cell colonies were stained with Methylene blue after growth in soft agar for 3 weeks. (**B**) GD25 cells were grown in suspension for 24 hours. Cells were fixed, stained with propidium iodine (PI) and analyzed by FACS. The increase in the G2/M cell population compared to the adherent control cells indicated that GD25 cells have functional cytokinesis block; (* represents *P* < 0.001 as evaluated by Student *t* test. The bars show the results from three independent experiments +/- SD. (**C**) GD25 M-cells were grown either in suspension or allowed to re-attach to tissue culture dishes. After 6 hours the cells were collected, fixed, stained with PI and analyzed by FACS. Cells in suspension retained the cytokinesis block, resulting in an increase in the G2/M population. However, attached cells finished cytokinesis, resulting in an increased G1 cell population. The bars show the results from three independent experiments +/- SD. (**D**) Phase contrast photographs of GD25-M cells taken before the FACS analysis described in (C). The same magnification (20 x) was used for the three pictures, and an enlargement of the two binucleated cells in the upper right corner of the middle frame (suspension culture) is shown in the inserted square. (**E**) Cell-doublets and single cells observed after 6 hours of suspension culture were counted and the percentages were plotted as a bar graph. The bars show the results from three independent experiments +/- SD; in each experiment at least 200 cells were counted. (**F**) GD25 cells in agar colonies completed cytokinesis. GD25 colonies isolated from soft agar at day 14 were trypsinized and subjected to FACS analysis. The histograms represent exponentially growing adherent cells (left histogram) and cells isolated from colonies grown in soft agar (right histogram). The distribution of cells in different cell cycle phases were quantified from three independent experiments and plotted as a bar graph +/- SD.

To characterize AIG, we initially used GD25 cells that form colonies in soft agar with high efficiency (approx. 25-30%, Figure 1A). GD25 cells are an SV40LT-immortalized mouse fibroblast-like cell line derived from an integrin β1 knockout embryonic stem cell [15]. GD25 cells have two main advantages that make them suitable for cell proliferation analysis in suspension culture. First, they have strongly reduced tendency to form cell aggregates, and therefore the influence of cell-cell contacts can be avoided. Second, when mitotic cells in suspension attempt to undergo cytokinesis, they acquire a bilobular-shaped structure. GD25 cells maintain this structure clearly visible for several hours, while in other cell lines the two un-separated daughter cell bodies rapidly makes close contact with each other and appear as an elongated structure in which it is difficult to determine whether the midbody is retained or not (see results below).

The functionality of suspension-induced cell cycle blocks in GD25 cells was tested by incubating the cells in suspension for 24 hours and then analyzing the cell cycle phase distribution by FACS. As expected, an increase in the G2/M cell population (from 19% to 34%) was observed in the suspension culture compared to the adherent cells (Figure 1B). This indicated that GD25 cells may have a functional cytokinesis block, however, the possibility that the cells were arrested in late G2 or M-phase could not be excluded from this experiment alone.

To confirm that GD25 cells were unable to complete cytokinesis in suspension culture, we synchronized cells in the M-phase using the “mitotic shake-off” method. Approximately 45-55% of the cells obtained by this method (M-cells) were in the G2/M-phase (Figure 1C). GD25 M-cells were either incubated in suspension or allowed to re-attach for 6 hours, and subsequently, the cells were subjected to FACS analysis. The re-attached M-cells completed cytokinesis and progressed to G1 phase during this period, as revealed by a decrease in the G2/M population (from 41% to 13%) and an increased G1 population compared to the freshly isolated M-cells. In suspension culture, however, the G2/M population was maintained (43%) while the G1 population decreased (Figure 1C).

As mentioned above, cytokinesis-blocked GD25 cells in suspension can be conveniently identified as dumbbell-shaped cell-doublets, i.e. two cells connected via a midbody, by phase-contrast microscopy (Figure 1D). Counting of cell-doublets and single cells in suspension cultures of GD25 M-cells revealed that the fraction of cell-doublets increased from 27% to 68% during the 6-hour incubation in suspension (Figure 1E). Furthermore, by time-lapse microscopy, no GD25 M-cells were observed to complete cytokinesis in suspension during 9 hours (Movie S1), while most cells completed this phase within 1-1.5 hour under adherent conditions after 6 hours in suspension. These data show that GD25 had a functional cytokinesis block in the absence of ECM adhesion signals.

Since the GD25 cells generated colonies in soft agar despite the cytokinesis block, we checked whether the agar colonies contained multinucleated cells. Colonies (14 days old) were isolated, treated with trypsin/EDTA, and the obtained cell suspensions were subjected to FACS analysis. These cells had similar FACS profiles as exponentially growing control cells (Figure 1F), suggesting that cells in soft agar managed to complete cytokinesis after extended periods in suspension by an unknown mechanism.

### Cells in suspension progressed to abscission stage of cytokinesis

**Figure 2:**
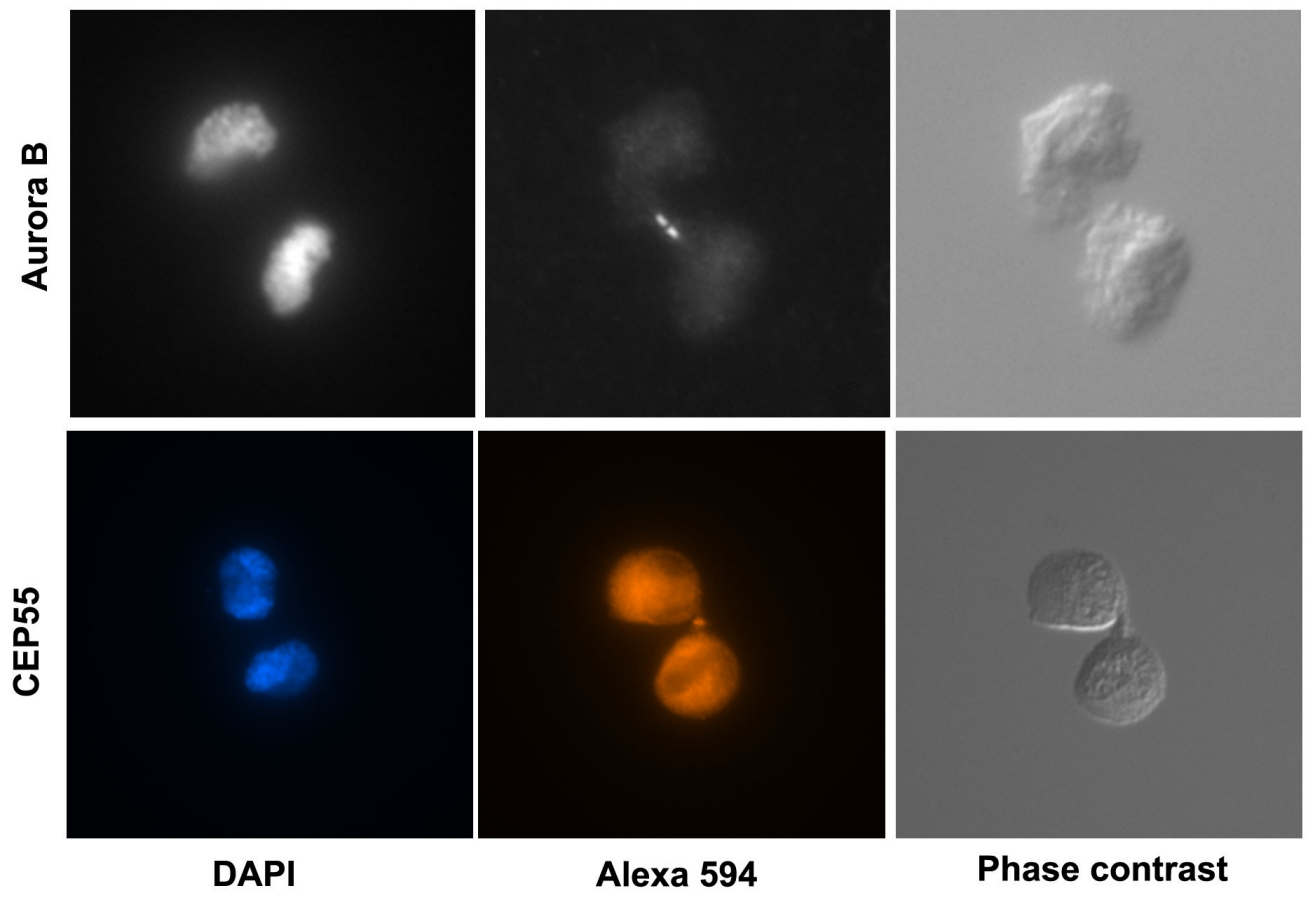
Cytokinesis proceeds to late stages in suspension culture. GD25 M-cells were incubated in suspension for 1.5 hours and then immuno-stained for aurora B and CEP55 as described in the Method section.

The dumbbell-shaped cell-doublets had distinct nuclei with de-condensed chromosomes and had thus passed the M-phase (see DAPI stained nuclei in e.g. Figure 2). To investigate at which stage of cytokinesis cells were arrested, we analyzed several markers of cytokinesis progression in GD25 M-cells kept in suspension for 1.5 h. During this time period, M-phase cells progressed to cytokinesis in suspension. As shown in Figure 2, the cytokinesis process reached to the stage where aurora B is associated to both sides of the midbody, and it even proceeded further to the association of CEP 55 with the midbody [16]. The suspension-induced cytokinesis block therefore concerned the final steps of the abscission process.

### Cells proceed in the cell cycle in spite of suspension-induced cytokinesis block

**Figure 3:**
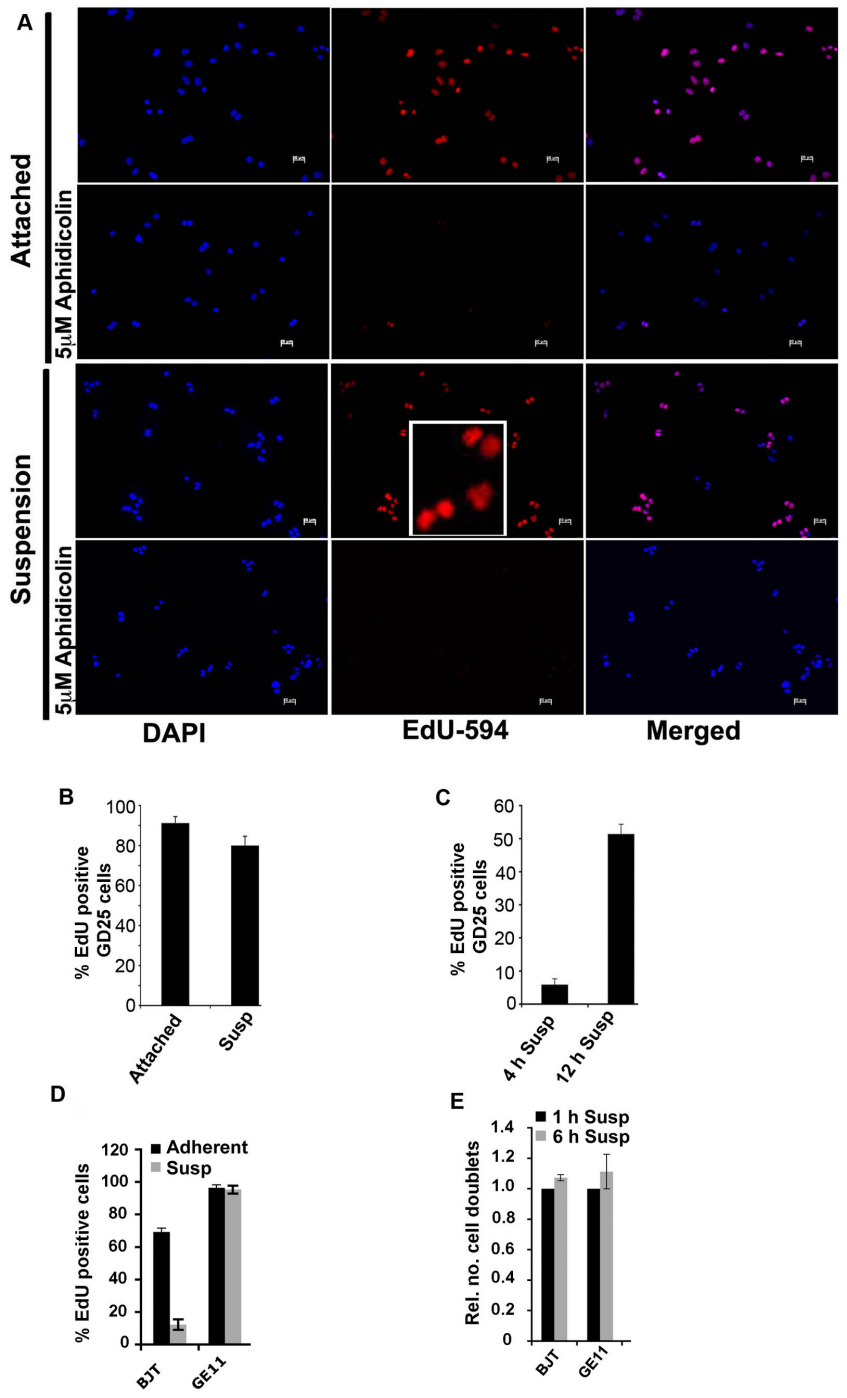
Analysis of cell cycle progression in suspension culture of selected cells lines. (**A**) GD25 M-cells kept in suspension or allowed to re-attach were incubated with 2.5 µM EdU for 16 hours in the presence or absence of aphidicolin. Both the adherent cells and the cells in suspension managed to proceed to S-phase (incorporated EdU). An enlargement of two EdU-stained binucleted cells in the lower right corner of the suspension culture frame is shown in the inserted square. (**B**) EdU positive cells from experiments described in (A) were counted. Almost equal numbers of cells incorporated EdU in both culture conditions. (**C**) GD25 M-cells were pulse-labeled for 60 min with 10 µM EdU after 4 hours or 12 hours in suspension culture, and the EdU positive cells were counted. (**D**) The indicated cell lines were analyzed for their ability to proceed into S-phase in suspension. Exponentially growing cells were trypsinized and either kept in suspension or allowed to adhere. After 3 hours, 2.5 µM EdU was added and the cells were incubated for 24 hours. Subsequently, the cells were fixed and the percentages of EdU positive cells were determined. (**E**) The indicated cell lines were analyzed for their ability to complete cytokinesis in suspension. M-cells isolated by the mitotic shake-off method were incubated in suspension for 1 and 6 hours, respectively, and cytokinesis block was determined as described in Materials and Methods. The bars in (B) - (E) show the results from three independent experiments +/- SD.

A small increase in the number of cells with >4N DNA content after 24 in suspension culture (Figure 1B) suggested that a new round of the cell cycle started even though the cytokinesis was not completed. To directly investigate whether the cells progressed to the S-phase in suspension culture, GD25 M-cells were incubated with 2.5 µM of the thymidine analog EdU for 16 hours (one generation time for adherent GD25 cells [17]). Almost equal numbers of cells (>80%) incorporated EdU in adherent and suspension cultures during this period, demonstrating that the cells entered S-phase under both conditions (Figure 3A, B). Treatment with aphidicolin (an inhibitor of DNA synthesis) essentially abolished the staining, validating that the EdU staining was due to DNA replication (Figure 3A). The results show that GD25 cells initiated a new round of the cell cycle although the cytokinesis was not completed and that they lacked a suspension-induced G1 block. Pulse-labeling of GD25 M-cells for 60 min with 10 µM EdU after 4 hours or 12 hours in suspension culture resulted in ~6% and 52% EdU positive cells, respectively (Figure 3C). The EdU positive dumbbell-shaped cells at the 12-hour time point must represent cytokinesis-blocked cells that had progressed to S-phase since the cells were pulse-labeled during a period (60 min) too short for any cells in the “shake-off“ population that might have been in the S-phase to pass through the G2 and M phases and reach the cytokinesis; the conclusion was further proven by the low number of labeled cells present at the 4-hour time point. From these experiments we concluded that GD25 cells were able to progress in the cell cycle despite the suspension-induced block of abscission. Additionally, blocking of cytokinesis by cytochalasin D treatment did not inhibit EdU incorporation in GD25 M-cells, neither in adherent (data not shown) nor in suspension cultures (Figure S1), further confirming that cytokinesis failure does not stop cell cycle progression in GD25 cells.

Several other cell lines that exhibited suspension-induced cytokinesis block but lacked G1 block, including NIH-3T3 and GE11, were similar to GD25 cells found to form colonies in soft agar (Table 1). The analysis was performed in the same way as for GD25 as illustrated for GE11 in Figure 3D, E. In contrast, BJT cells had a functional G1 block and therefore could not generate colonies in soft agar (Figure 3D, E, Table 1), as reported for other cell lines with non-suppressed G1 checkpoint.

**Table 1:** A summary of the colony growth in soft agar and the presence of suspension-induced cytokinesis and G1 blocks for the indicated cell lines.

Cell line	Soft agar colonies	Cytokinesis block in susp.	G1-block in susp.
BJT^*^	No	Yes	Yes
NIH-3T3^**^	Yes	Yes	No
GE11^**^	Yes	Yes	No
GD25^**^	Yes	Yes	No
GD25β1^**^	Yes	Yes	No

^*^ Immortalized with hTERT

^**^ Transformed with SV40 large T antigen

### Fibronectin is required for agar colony formation of cells with suspension-induced cytokinesis block

The above analyses strongly indicated that the GD25 cells were able to complete cytokinesis in suspension only after prolonged culture times. Previous reports suggest that ECM components enhance colony formation in soft agar [7,18,19] and ECM-integrin signaling has been shown to be required to complete cytokinesis [4,14]. In particular, FN has been suggested to regulate cytokinesis [19], AIG [7,18], and the assembly of ECMs [20,21]. Therefore, we investigated the possibility that GD25 cells during soft agar culture managed to assemble an ECM that provided sufficient signals to complete the cytokinesis. Soft agar assays in the presence of an RGD peptide (GRGDS) that competitively inhibits the interaction of several integrins with FN and vitronectin [22], did not prevent colony formation, but it delayed their initial growth (data not shown). Since the effect of the added RGD peptide was incomplete, conceivably due to inefficient competition during long-term experiments, more specific approaches were applied

**Figure 4:**
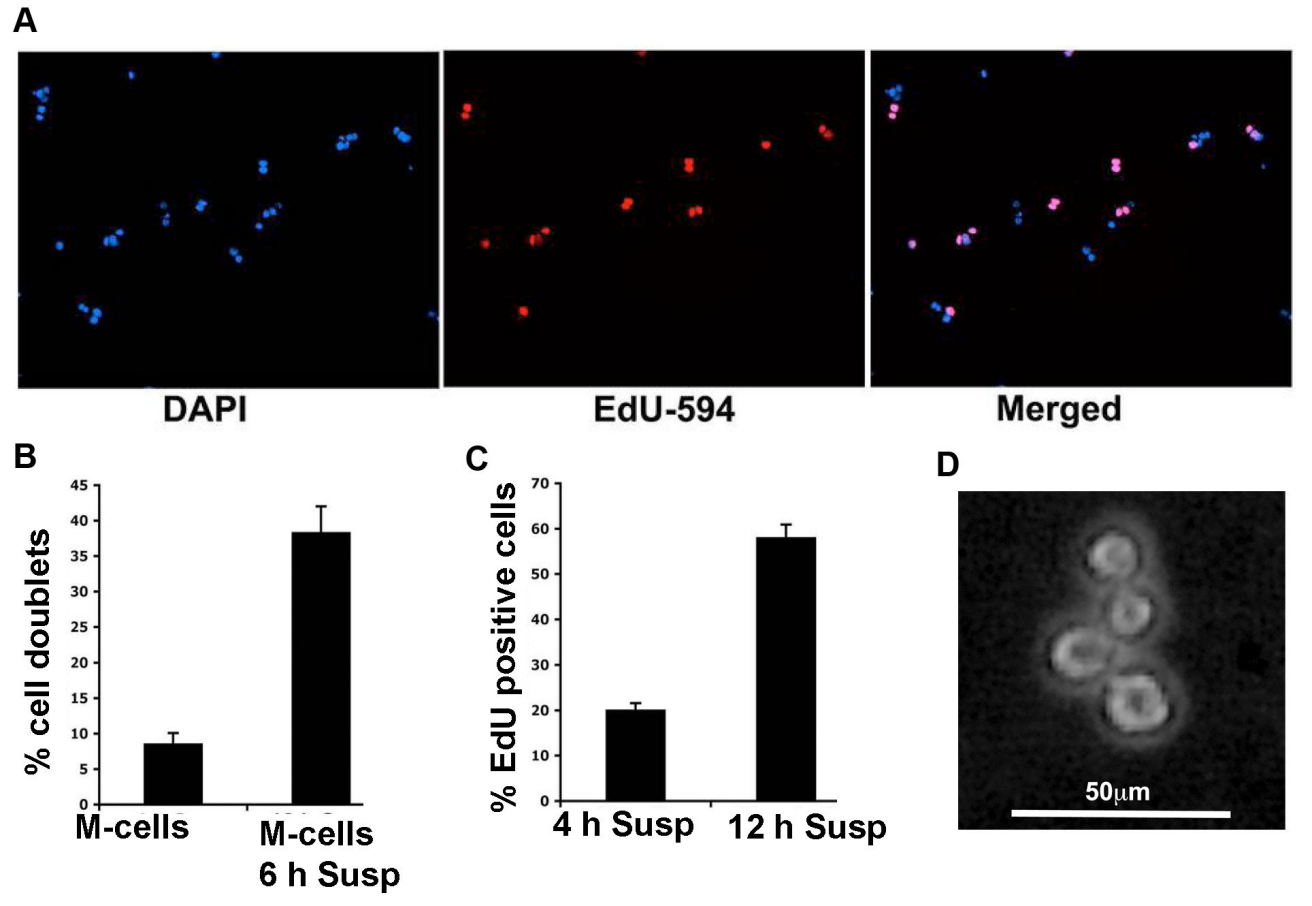
Analysis of suspension-induced G1 and cytokinesis blocks in FN deficient 4D cells. (**A**) Photographs of 4D M-cells grown in suspension for 12 hours and then incubated with 10 µM EdU for 60 min. (**B**) 4D cells in suspension retained the cytokinesis block. The number of 4D cell-doublets and single cells were counted directly after isolation of M-cells and after 6 hours suspension culture. (**C**) Cytokinesis-blocked 4D cells in suspension progressed in the cell cycle. 4D M-cells were EdU-labeled for 60 min with 10 µM EdU after 4 hours or 12 hours in suspension culture, and the number of labeled cells was counted. (**D**) Photograph of a 4D M-cell grown in suspension (methylcellulose) for 48 hours and then trypsinized as described in Materials and methods to reveal the connection between cell bodies. The bars in (B) and (C) show the results from three independent experiments +/- SD.

To directly investigate the role of FN in AIG, we used the SV40LT-immortalized FN^-/-^ cell line 4D [23]. These cells were found to possess a functional suspension-induced cytokinesis block but were still able to progress to S-phase in suspension culture (Figure 4A–C), similar to GD25. Interestingly, 4D cells could not generate any colonies in the soft agar assay, only occasionally few colonies were seen (Table 2). Careful analysis revealed small aggregates of cells at early time points (first week), however, these aggregates did not grow. Time-lapse movies of 4D M-cells plated in methylcellulose-containing medium in non-adherent plates showed that the cells managed to complete initial rounds of the cell cycle (data not shown). After 48 hours in methylcellulose, the cell aggregates were isolated and trypsinzed to investigate whether they had completed cytokinesis. As expected, cells-bodies connected with a thin tubular structure were often seen (Figure 4D). These results showed that the inability of 4D cells to form colonies in soft agar was not due to G1 checkpoint activation.

**Table 2:** Colony growth dependent on exogenously added FN.

Number of 4D cell colonies		
	No FN	Added FN
Exp. 1	2	59
Exp. 2	4	88
Exp. 3	0	48

4D cells (FN^-/-^) were cultured in soft agar with and without supplementation of exogenous FN. The numbers of colonies formed after 4 weeks are shown.

The possibility that the failure to form colonies was linked to the absence of FN synthesis was tested by supplementation of the culture medium with isolated plasma FN. Intriguingly, addition of exogenous FN rescued the ability of 4D cells to form colonies in agar, although the colonies grow slowly (Table 2). To confirm this result, we used another FN^-/-^ cell line generated by the cre-LoxP system and the corresponding FN-expressing cell line (FN^fl/fl^) as control [24]. These FN^-/-^ cells did form some colonies, however, the number was 7-9 fold lower than for the FN^fl/fl^ cells (Table 3). Both cell lines had functional suspension-induced cytokinesis block and dysfunctional G1 block (Figure S2).

**Table 3:** Colony growth dependent on endogenous FN **synthesis**.

Number colonies		
	FN^-/-^ MEF	FN^fl/fl^ MEF
Exp. 1	63	552
Exp. 2	81	572

FN^-/-^ MEF and FN^fl/fl^ MEF were grown in soft agar for 3 weeks and the numbers of colonies formed are shown.

**Figure 5:**
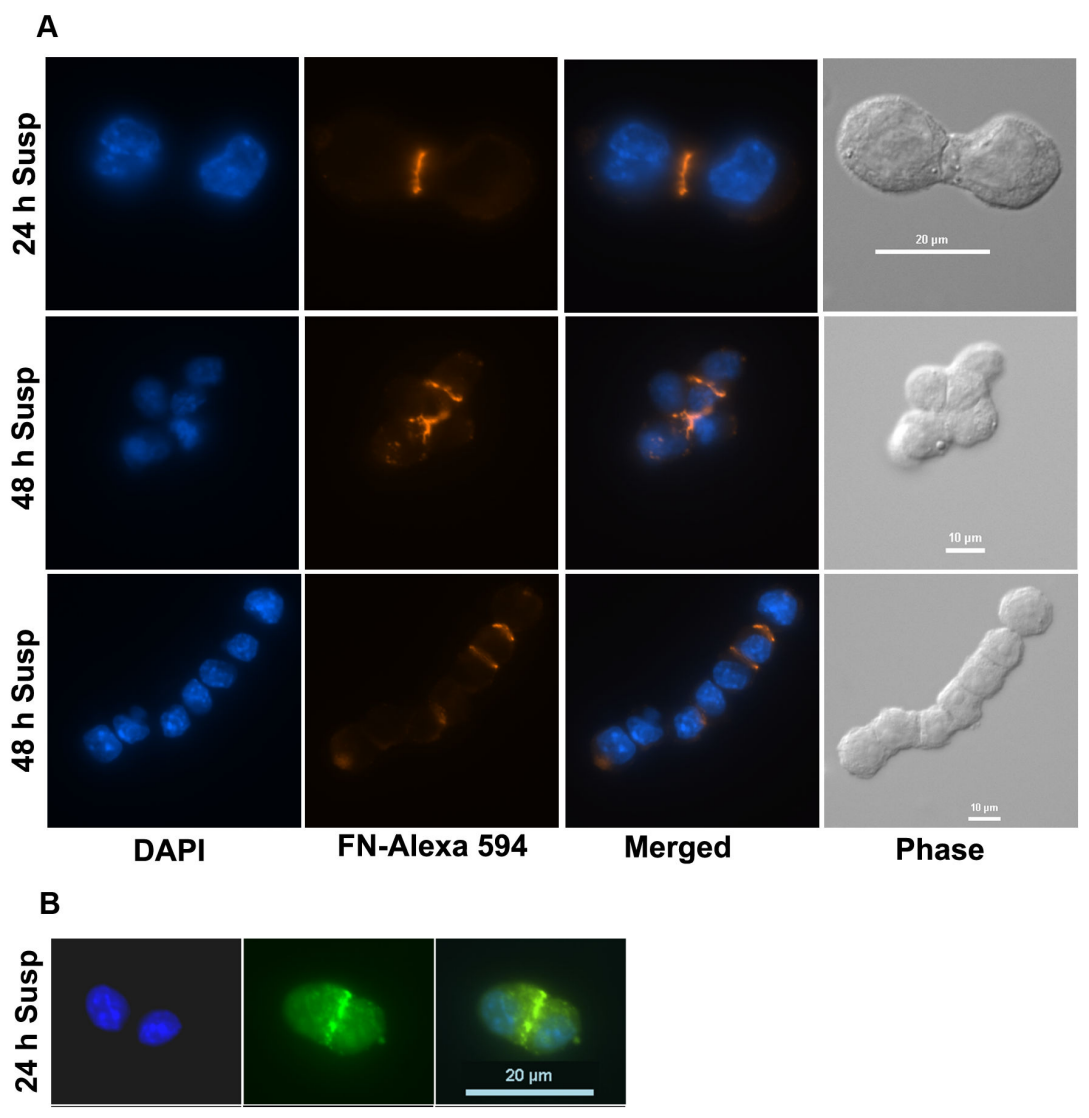
Deposition of FN at intussusceptions of bi- and multilobular cells during suspension culture. (**A**) GD25 M-cells grown in 0.8% methylcellulose for 24 or 48 hours were immuno-stained for FN. (**B**) GD25 M-cells were grown in 0.8% methylcellulose in the presence of 250 µg/ml FITC-labeled FN for 24 hours. The cells were then washed with PBS and fixed with 4% formaldehyde. The cells were analyzed in a Nikon fluorescence microscope. The panels show the FN deposition at the two-, four- and 8-lobular cellbody stages. During parts of the cell cycle the multilobular cells were folded (C, middle panel), whereas elongated structures were seen during the karyokinesis phases.

The above results showed that FN had a critical role in AIG. To be functionally active and to induce signals, the soluble FN needs to be deposited on the cell surface. Therefore, we investigated whether FN assembly actually occurred on cells in suspension by two methods: 1) Immunofluorescent staining of GD25 M-cells with FN antibodies after suspension culture in methylcellulose for 24 or 48 hours revealed that FN indeed was deposited on the cells in suspension. The staining was localized mainly in the intussusceptions formed between the cell bodies of the cytokinesis-blocked cells (Figure 5A). 2) Similar results were obtained after incubation of GD25 M-cells with FITC-labeled plasma FN for 24 hours (Figure 5B). Notably, the intussusceptions and the cell bodies formed after the second and third rounds of mitosis in suspension (4 and 8 nuclei, respectively) were initially arranged linearly, showing that the orientation of the mitotic spindle was determined in a specific manner (Movie S2). Later on after each karyokinesis, the cell-lobes formed contact with each other.

Since colony formation of cells that retain functional suspension-induced cytokinesis block was dependent on ECM-integrin interactions, we termed it “pseudo AIG”.

### Uncoupling of cytokinesis from mitosis

**Figure 6:**
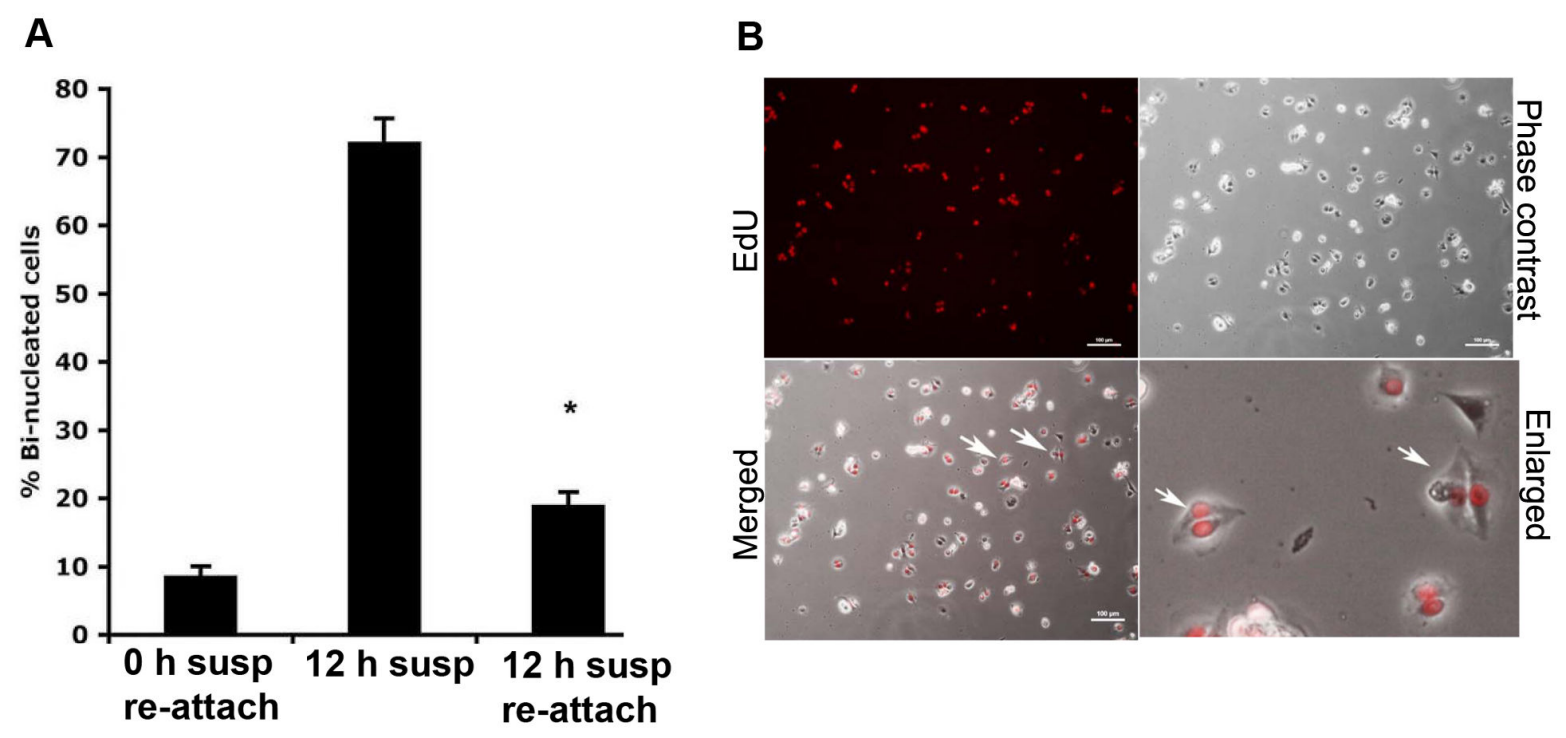
Re-attachment induces cytokinesis of suspension-cultured GD25 cells. (**A**) Re-attachment generated elevated numbers of binucleated cells. GD25 M-cells were plated in tissue culture flask for 6 hours directly after isolation (left bar) or after 12 hours in suspension (right bar). The number of cell-doublets (cells in suspension culture for 12 hours, middle bar) or binucleated cells (attached cells) was counted (more than 200 cells of each category in all sets of experiment). The average of three experiments +/- SD is shown, and a statistically significant increase in the number of binucleated cells were observed when cells were incubated in suspension prior to adhesion (compared to immediately re-plated cells) as evaluated by Student *t* test (*, *P* < 0.001). (**B**) Completion of cytokinesis (abscission) occurred uncoordinated with the M-phase. GD25 M-cells, grown in suspension for 12 hours, were plated on vitronectin-coated coverslips in the presence of 10 µM EdU. Time-lapse movies were recorded for 1.5 hours and during this time period, most cells completed cytokinesis. Cells were fixed and analyzed for EdU incorporation. Some of the cells that completed cytokinesis also incorporated EdU, suggesting that cytokinesis completion can be uncoupled from mitosis.

To test whether the cytokinesis-blocked cells really could complete the abscission at a later stage upon renewed ECM-induced signaling, GD25 M-cells were incubated in suspension for 12 hours and then allowed to re-attach. Time-lapse monitoring and binucleation analysis (Figure 6A) demonstrated that a high proportion (>70%) of the binucleated cells retained the ability to complete the cytokinesis process. Since our previous analysis showed that cells progressed in the cell cycle while retaining the cytokinesis block, we investigated the possibility that cells may be in other stages of the cell cycle than M-phase when completing the cytokinesis. GD25 M-cells were incubated in suspension for 12 hours, and then 10 µM EdU was added and the cells were allowed to re-attach to vitronectin-coated coverslips. After time-lapse movie recordings for 1.5 hour, the cells were fixed and analyzed for EdU incorporation. Most cells finished cytokinesis during this adhesion period and some of them also had incorporated EdU, demonstrating that these cells were in S-phase (Figure 6B). These analyses show that the cells retained the ability to complete cytokinesis, and that it could occur uncoordinated with the M-phase. However, an increased frequency of cytokinesis failures occurred since ~19% of the re-attached cells formed binucleated cells, compared to 9% binucleated cells present in re-adhered freshly isolated M-cells (Figure 6A).

## Discussion

**Figure 7:**
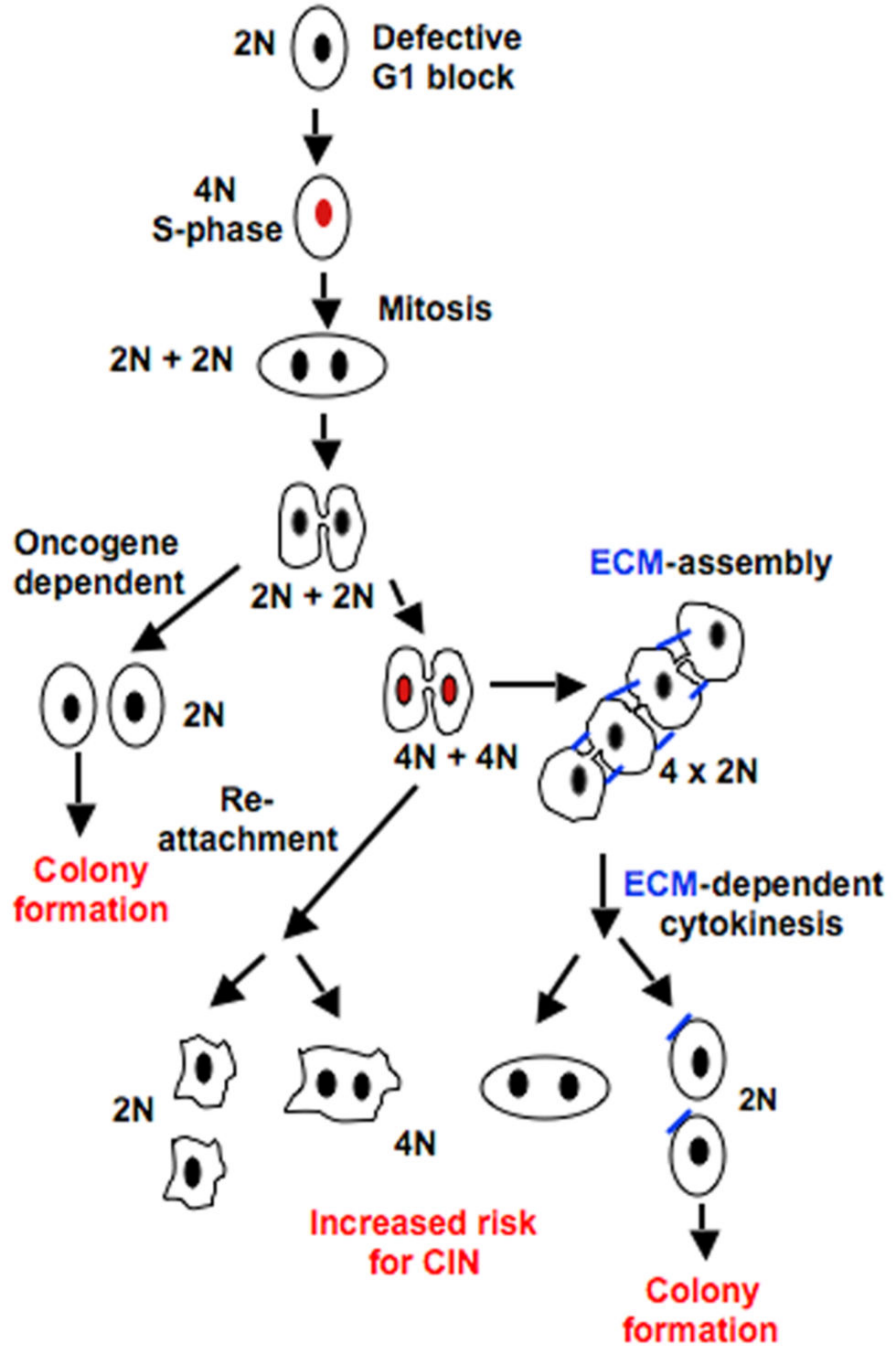
Schematic model for colony growth in suspension and the formation of binucleated cells. When the G1 checkpoint is suppressed, detached cells progress in the cell cycle and reach the cytokinesis phase. Cells expressing oncogenes (e.g. overactive Ras) complete the cytokinesis and proliferate in agar. Alternatively, cells can finish cytokinesis after renewed ECM-integrin signals by adhesion or FN assembly. FN deposition on the cell surface involves exposure of FN–FN binding sites by mechanical stretching of the protein, which occurs normally between neighboring cells and is dependent on the stiffness of the support. Consequently, it is very inefficient on single cells in suspension. However, the multilobular structure of cytokinesis-blocked cells after two or more rounds of the cell cycle promotes the assembly of secreted FN. The incidence of binucleation is high if cells enter the cell cycle without completing cytokinesis. Black nuclei represent G1 phase; red nuclei represent S phase; rounded morphology of cells represent non-adherent cells.

The soft agar assay is an extensively used method to define the transformed nature of cells. Our results clarify important underlying mechanisms that regulate growth in soft agar, and based on the data, we propose that two alternative mechanisms support AIG. According to our model (Figure 7), G1 phase de-regulation is an essential feature for AIG. If this prerequisite is met, the suspension-induced cytokinesis-block can then be resolved either by signals driven by oncogenes or by signals from ECM-integrin interactions (pseudo AIG). An example of the first mechanism is AIG relying on signals generated by Ras mutations [4], which otherwise are induced downstream of integrins in normal cells under adherent conditions. Activation of p110α-containing PI3Ks is known to be one of these required signals induced by integrins in a Ras-dependent manner [25–27], but it is yet unclear if it is involved in the cytokinesis step, or if it contributes to colony growth primarily by the anti-apoptotic functions of PI3K-activated AKT. Constitutively active HRas was reported to overcome the inhibiting effect of the drug Y27632 on cytokinesis in adherent cells, which may suggest that HRas activates targets downstream of ROCK [4]. However, the regulation of cytokinesis by Ras proteins is still an important unresolved issue.

The alternative mechanism supporting AIG involves ECM-deposition on cells in suspension culture. Polymerization of secreted FN is a key step in the assembly of ECM since the protein has binding sites for collagens and proteoglycans, and deposition of collagen I and III by fibroblasts has been shown to require FN [28,29]. FN polymerization occurs after binding to integrins (mainly α5β1, and also αvβ3 [30,31]) and is strongly promoted by RhoA-dependent mechanical tension through the RhoA-ROCK-myosin II-actin-talin-integrin-FN linkage [30,32–34]. The stretching of FN molecules bridged between cells, or between one cell and a solid support, causes exposure of cryptic FN–FN binding sites required for the polymerization [35]. FN deposition therefore occurs primarily between neighboring cells [30,31] and is influenced by the stiffness of the surrounding support [36] and, accordingly, it is very inefficient on single cells in suspension. However, the lobular structure of cells in suspension, formed as a consequence of continued cell cycle progression in the absence of complete cytokinesis, apparently creates conditions that promote deposition of FN (Figure 6) and possibly other secreted ECM proteins. The process resembles the FN assembly that normally occurs between neighboring cells, and the initially slow rate is presumably due to weak tension forces in the absence of anchorage of the cells to a stiff support. Nevertheless, ultimately enough ECM will be assembled to bring cytokinesis to completion. These results provide an explanation to previous reports where TGFβ, in the presence of other growth factors, was found to promote AIG of some cell lines through increased FN synthesis [7,18]. The S, G2 and M phases have been reported to proceed normally in suspension cultures of G1-phase synchronized cells [4]. However, progression in the next round of the cell cycle of detached, cytokinesis-blocked cells has not previously been investigated. Our study revealed that the cell cycle continued in this situation, although it was delayed and the phases were heterogeneous in duration (data not shown). Similar observations have previously been reported for adherent cells treated with drugs to block cytokinesis [37]. In long-term movies (Movie S2), GD25 cells were initially seen to adopt elongated bead-like structures of 4 cell bodies after undergoing a second round of karyokinesis in suspension culture, which later often were bent and formed tight structures. This orientation of the mitotic spindles implies that each cellbody arranged one centrosome close to the midbody. Since FN was deposited close to the midbodies, our observations are consistent with the reported data showing that the localization of centrosomes is guided by the extracellular matrix [38]. However, further investigations are needed to clarify what signaling events regulate centrosome localization.

What is the role of AIG in cancer? While the ability to survive and proliferate anchorage-independently has been convincingly demonstrated to be of critical importance for metastatic spreading via the circulation [39], it has not been clear whether this ability have any significance for tumor initiation and progression. Pseudo anchorage-independent cells are not directly tumorigenic, but our data indicate that they have the potential to generate tumors under certain conditions, which are dependent on the local environment of the host. In agreement with previous observations [40], we found that periods with cells in suspension followed by renewed integrin signaling increased the formation of bi/multinucleated cells, which is known to cause chromosomal instability and subsequent selection of malignant cells [41]. In experimental situations, the generation of bi-nucleated cells would be dependent on the ability of the transplanted cells to survive during the period they would lack integrin signals. For non-experimental tumorigenesis in humans, the increased risk for binucleation and CIN after one or more rounds of suspension-adhesion cycles may also be a significant factor. It may promote tumor progression upon re-attachment of detached tumor cells locally (e.g. in body cavities) [42,43] or at distant sites after transportation via the circulation. We further hypothesize that pseudo AIG may cause tumor *initiation* during persistent inflammation. Although yet not extensively studied, it has been shown that detachment of cells into body cavities occur during inflammation as a result of protease activity [44]. Additionally, excessive cytokine stimulation during inflammation, or virus infections, may suppress the G1 checkpoint and thereby set the stage for increased risk of tumor initiation through pseudo AIG as illustrated in Figure 7. Thus, the identification of pseudo AIG offers a new direction to study the connection between chronic inflammation and cancer.

## Material and Methods

### Cell culture

The following cell lines were maintained in DMEM supplemented with 10% fetal bovine serum and antibiotics (complete medium) as described earlier [45]: GD25 (integrin β1-deficient mouse fibroblast) [15], GD25β1 (GD25 stably transfected with mouse β1 cDNA) [31], 4D (FN-deficient mouse fibroblast) [23], LoxP/Cre-generated FN^-/-^ MEF cell line and the corresponding β1 expressing control cell line with floxed β1 gene (FN^fl/fl^) [24,46], BJT cells (telomerase-immortalized human fibroblast) [47], NIH-3T3/13C7 cells (RIKEN, Cell Bank, # RCB0057). Cell synchronization in M-phase was done by the shake-off method using flasks containing exponentially growing cells. Suspension culture of cells was performed in dishes (Bacterial grade 10 cm dishes; Sarstedt, Sweden) coated with 1% Pluronic F 108NF (D-BASE, USA) or 1% heat-treated BSA (Catalog No. 10735094001, Roche Diagnostics, Indianapolis, USA). To avoid cell aggregation during suspension culture, the cells were seeded at low density (10 000 cells/ml) and exposed to occasional careful pipetting for short-term suspension culture. For long-term suspension culture (more that 20 h), 1.2% methylcellulose medium (Catalog No. M0512, Sigma-Aldrich, USA) in complete medium was used. Coverslips coated with 10 µg/ml vitronectin were used for reattachment experiments. Aphidicolin (Catalog No. A0781, Sigma-Aldrich, USA) and cytochalasin D (Catalog No. sc-201442, Santa Cruz Biotechnology, USA) was used as described in the figure legends.

### Soft-agar assay

Soft agar assay was done in 6 cm dishes. A bottom layer of 4 ml, 0.8% agarose (Lonza, Catalog No. 50 000) in complete medium, was made. On top, a layer containing 10 000 cells mixed in 3 ml 0.35% agar (Difco, Catalog No. 214220, Becton, Dickinson and Company, Spark, USA) in complete medium was spread. After 2 days incubation, another layer of top agar in complete medium was added. Colonies were visualized by staining with 0.005% Methylene blue and photographs were taken in a dissection microscope. To block interactions of RGD-recognizing integrins with ECM proteins, 100 µg/ml G**RGD**S (gly–arg–gly–asp–ser; Bachem, Germany) peptide was added together with the cells. The role of FN in colony growth was analyzed by seeding 100 000-200 000 4D cells in 75 cm^3^ flasks in the presence or absence of FN (250 µg/ml; purified from human blood plasma) [48] in complete medium. After 42 hours, the cells were harvested by scrapping and subjected to soft agar analysis in the presence or absence of 250 µg/ml FN.

### FACS analysis

FACS analysis was done as reported earlier [49]. Briefly, cells were washed with PBS and fixed in 80% methanol. Cells were stained with propidium iodine (PI) (50 µg/ml PI, 20 µg/ml RNase A, and 0.1% Triton X-100 in PBS) and analyzed in a FACS (BD FACSAria, BD Biosciences) using FACSDiva software. To avoid cell aggregates, the FACS samples were passed through a 50 µm filter (Cat No. 340631, BD Biosciences, San Jose, CA, USA).

### EdU incorporation analysis

EdU detection was done according to the manufacturer instruction (Click-iT^TM^ EdU Imaging Kit, Catalog No. C10084, Invitrogen Molecular Probes, Eugene, Oregon, USA). Briefly, long-term labeling of cells was done in the presence of 2.5 µm EdU while pulse labeling was done in the presence of 10 µm EdU as described in the figure legends. Cells were fixed with 4% formaldehyde and analyzed for EdU incorporation.

### Time-lapse movie

Time-lapse movies were recorded as described [49]. Briefly, cells in suitable Petri dishes were placed in the motorized stage of an Axiovert 200M-inverted microscope (Carl Zeiss) equipped with a cell culture chamber having constant supply of humidified 5% CO2 and temperature control.

### Labeling of FN with FITC

FN (1 mg) was labeled with FITC (4 mg) in 1.2 ml sodium bicarbonate buffer (pH 9.5) overnight at 4° C. Labeled FN was purified by PD-10 column chromatography (Catalog No. 17-0851-01, GE healthcare).

### Immuno-staining

Immuno-staining of GD25 cells for surface-associated FN was performed after fixation in 4% paraformaldehyde for 10 min at room. The fixed samples were incubated with 5% BSA in PBS for 1 hour and subsequently incubated at 4° C overnight with an anti-FN antiserum [50]. After washing with PBS containing 0.05% Tween-20 and 0.5% BSA, the samples were incubated with secondary antibodies labeled with Alexa-488 for 1 h at room temperature. Then the samples were washed and counter-stained with DAPI. The immuno-stainings for aurora B and CEP55 were performed in the same way except that the cells were permeabilized for 10 min with 0.5% Triton X-100 and 0.05% Tween-20 in PBS after the fixation step, and the secondary antibody was labeled with Alexa-594.

### Determination of suspension-induced cytokinesis-block using trypsin-EDTA treatment

For many cell types it is difficult to ascertain whether cytokinesis in suspension becomes completed or not, since they rapidly form close cell-cell contacts in both cases. To determine if suspension culture induced blockade of cytokinesis in different cell lines we developed a trypsin/EDTA treatment method. This method is based on our observation that incubation with trypsin/EDTA does not disrupt the midbody linking two daughter cells, but removes other cell contacts. Mitotic cells, collected by the mitotic shake-off method, were incubated in suspension culture for 1 hour and 6 hours, respectively. Subsequently, the cells were incubated with 0.25% trypsin/1mM EDTA for 5-10 minutes, and cell doublets and single cells were counted. The percentage of cell doublets at 1 hour was set as 1, and the percentage of cell doublets at the 6 hours time point was calculated in relation to the 1-hour time point.

## Supporting Information

Figure S1 **GD25 cells enter cell cycle in spite of cytokinesis failure due to cytochalasin D treatment.** (**A**) GD25-M cells in suspension were treated for 2 hours with 1 µM cytochalasin D. Subsequently, the drug was washed away and the cells were allowed to reattach to a culture dish for 10 hours. The analysis of the number of binucleated cells showed that cytochalasin D treatment efficiently blocked cytokinesis. The bars show the results from three independent experiments +/- SD. (B) GD25-M cells in suspension were treated for 2 hours with 1 µM cytochalasin D. Subsequently, the drug was washed away and the cells were kept in suspension for 11 hours followed by 1h in suspension in the presence of 10 µM EdU. Cells were fixed and analyzed for EdU incorporation. Many binucleated cells (indicated by arrow) incorporated EdU. The total percentage of EdU positive cells was approximately 56%, and among these approximately 73% cells were binucleated. Note that cytochalasin D treated cells did not form a cleavage furrow.
(TIF)

Figure S2 **Analysis of cell cycle progression in suspension culture of FN^-/-^ MEF and FN^fl/fl^ MEF.** (**A**) The cell lines were analyzed for their ability to proceed into S-phase in suspension. Exponentially growing cells were trypsinized and either kept in suspension or allowed to adhere. After 3 hours, 2.5 µM EdU was added and the cells were incubated for 24 hours. Subsequently, the cells were fixed and the percentages of EdU positive cells were determined. (**B**) The cell lines were analyzed for their ability to complete cytokinesis in suspension. M-cells isolated by the mitotic shake-off method were incubated in suspension for 1 and 6 hours, respectively, and cytokinesis block was determined as described in Materials and Methods. The bars in (A) and (B) show the results from three independent experiments +/- SD.
(TIF)

Movie S1 **Time-lapse movie of GD25 M-cells, showing that the cells are not able to complete cytokinesis in suspension culture.** Each frame of the movie was captured at an interval of 3 minutes. The total running time of the movie is 9 hours. The movie is played at a rate of 10 frames/sec.
(AVI)

Movie S2 **Time-lapse movie of GD25 M-cells showing the symmetry of cell bodies in two consecutive cell divisions in suspension**. M-cells have two nuclei (start of the movie recordings). The two cell lobes are not distinguishable because the presence of methylcellulose in the culture medium during the video recording promotes a close association between the lobes. During the subsequent karyokinesis, the cells arranged one of the centrosomes in proximity to the midbody zone, resulting in linear cellbody structures. Note that when cells enter the mitotic phase, they disrupt cell-cell contacts between neighboring cell bodies. Each frame of the movie was captured at an interval of 5 minutes. The total running time of the movie is 29 hours and it is played at a rate of 10 frames/sec.
(AVI)
