# Supplementary figures and images for: Fibronectin Assembly in the Crypts of Cytokinesis-Blocked Multilobular Cells Promotes Anchorage-Independent Growth
Source: PLoS One. 2013 Aug 12;8(8):e72933. doi: 10.1371/journal.pone.0072933 (PMC3741384; doi:10.1371/journal.pone.0072933)

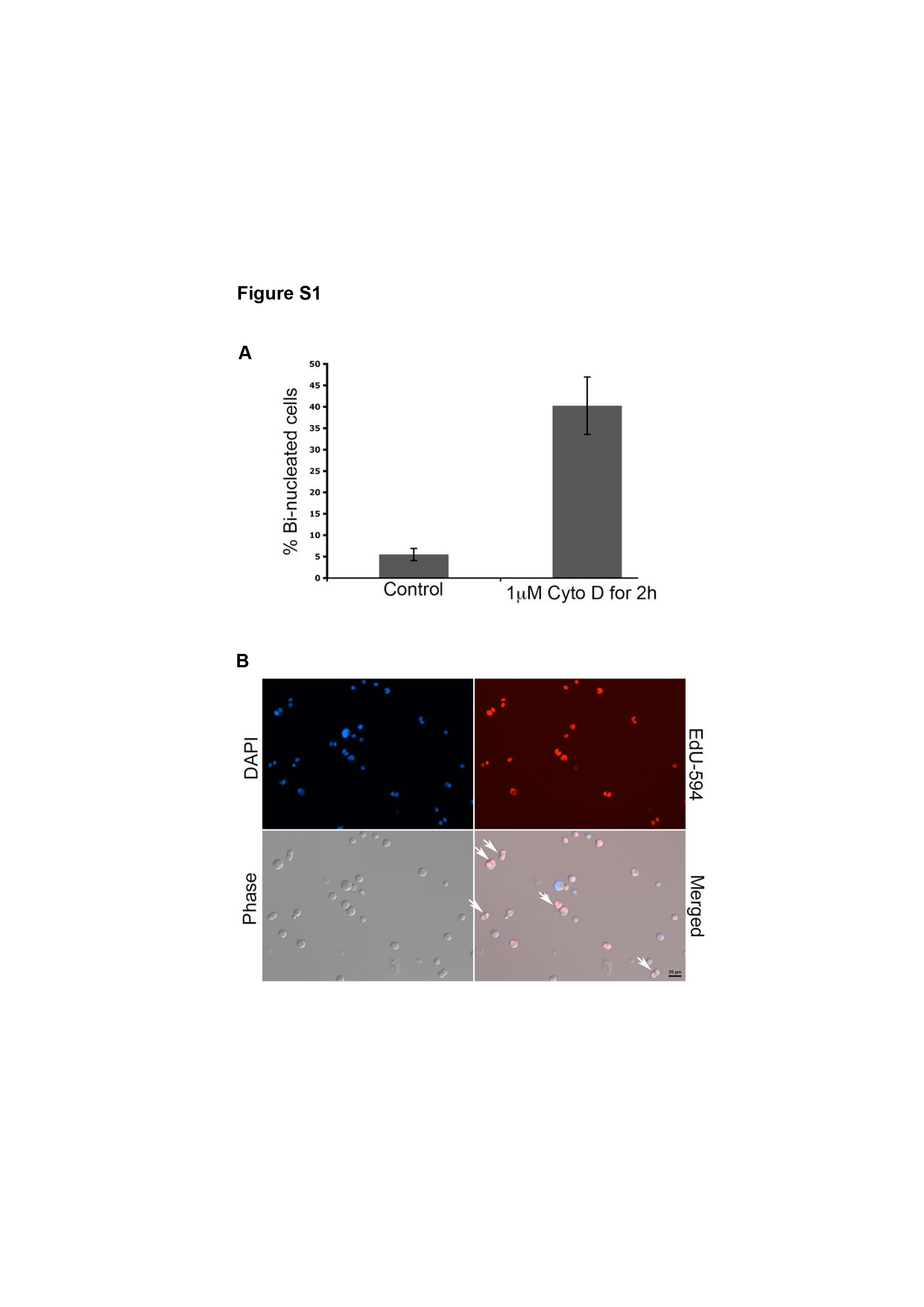

Supplement: Figure S1 — GD25 cells enter cell cycle in spite of cytokinesis failure due to cytochalasin D treatment. (A) GD25-M cells in suspension were treated for 2 hours with 1 µM cytochalasin D. Subsequently, the drug was washed away and the cells were allowed to reattach to a culture dish for 10 hours. The analysis of the number of binucleated cells showed that cytochalasin D treatment efficiently blocked cytokinesis. The bars show the results from three independent experiments +/- SD. (B) GD25-M cells in suspension were treated for 2 hours with 1 µM cytochalasin D. Subsequently, the drug was washed away and the cells were kept in suspension for 11 hours followed by 1h in suspension in the presence of 10 µM EdU. Cells were fixed and analyzed for EdU incorporation. Many binucleated cells (indicated by arrow) incorporated EdU. The total percentage of EdU positive cells was approximately 56%, and among these approximately 73% cells were binucleated. Note that cytochalasin D treated cells did not form a cleavage furrow. (TIF) [file pone.0072933.s001.tif]

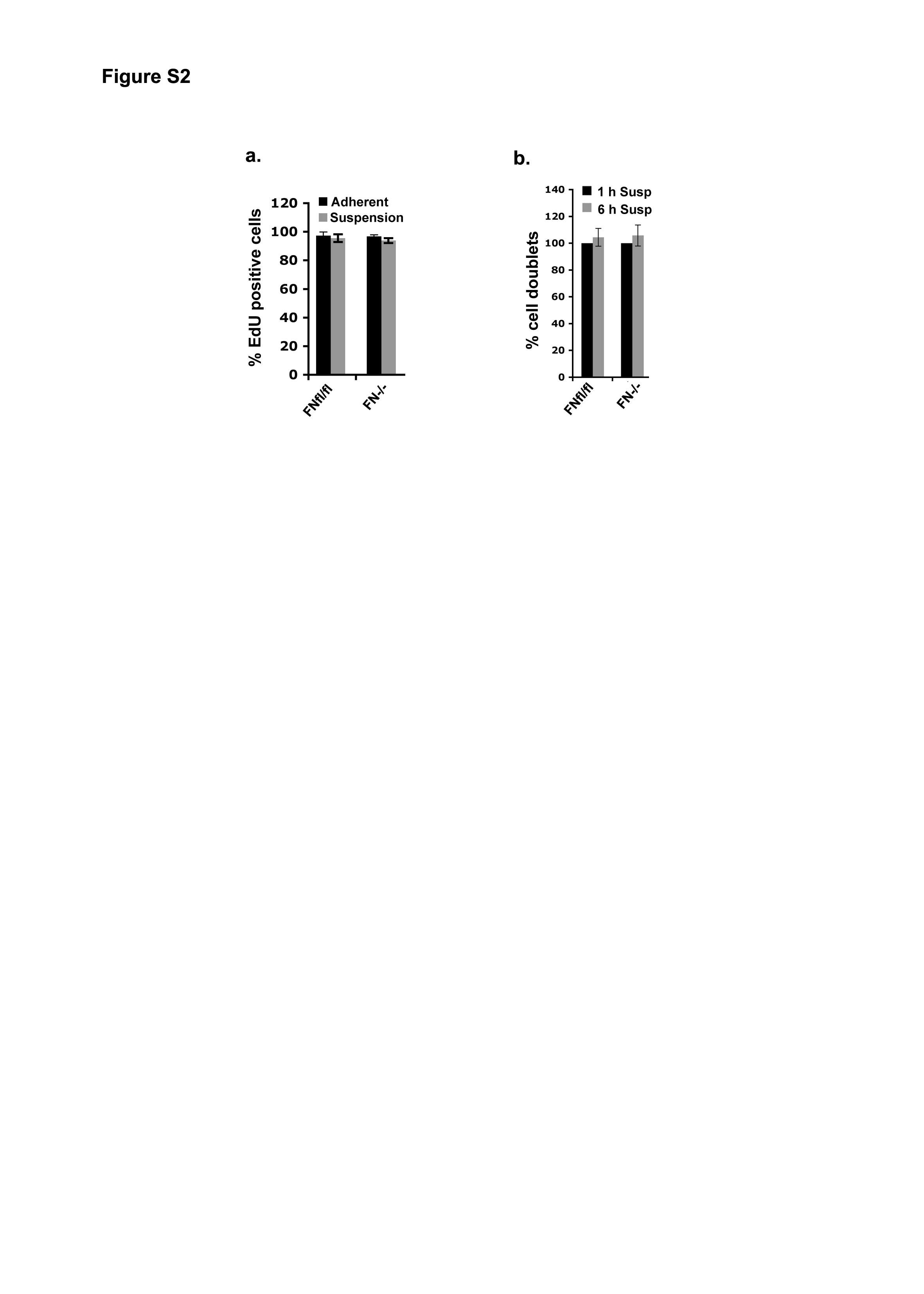

Supplement: Figure S2 — Analysis of cell cycle progression in suspension culture of FN-/- MEF and FNfl/fl MEF. (A) The cell lines were analyzed for their ability to proceed into S-phase in suspension. Exponentially growing cells were trypsinized and either kept in suspension or allowed to adhere. After 3 hours, 2.5 µM EdU was added and the cells were incubated for 24 hours. Subsequently, the cells were fixed and the percentages of EdU positive cells were determined. (B) The cell lines were analyzed for their ability to complete cytokinesis in suspension. M-cells isolated by the mitotic shake-off method were incubated in suspension for 1 and 6 hours, respectively, and cytokinesis block was determined as described in Materials and Methods. The bars in (A) and (B) show the results from three independent experiments +/- SD. (TIF) [file pone.0072933.s002.tif]
